# Attitudes towards Freshly Made and Readily Prepared Texture-Modified Foods among Speech-Language Therapists, Dietitians, and Community-Dwelling Older Adults

**DOI:** 10.3390/foods11142157

**Published:** 2022-07-20

**Authors:** Xiaojing Sharon Wu, Anna Miles, Andrea Braakhuis

**Affiliations:** 1 Department of Nutrition, Faculty of Medical and Health Sciences, University of Auckland, Auckland 1023, New Zealand; a.braakhuis@auckland.ac.nz; 2 Department of Speech Science, School of Psychology, University of Auckland, Auckland 1023, New Zealand; a.miles@auckland.ac.nz

**Keywords:** texture-modified foods, dysphagia, consumer, hydrolysed meat, sous-vide commercial, attitudes, health professionals, mixed-methods

## Abstract

Texture-modified foods (TMFs) are recommended for patients suffering from swallowing difficulties. Given the increasing aging population, the use of TMFs is on the rise. Research to date has focused on the nutritional value, malnutrition indices and healthcare practices in relation to TMFs, but the perception of these diets from a patient and healthcare practitioner perspective has received less consideration. This study explored how currently available TMFs (including Soft & Bite-Sized, Minced & Moist, and puree) are perceived by key stakeholders. Four types of TMFs were consumer tested: freshly made TMFs following foodservice recipes and three types of readily prepared TMFs (commercially packaged, sous-vide and hydrolysed). The selected samples were tested through five focus groups (including nine dietitians, seven speech-language therapists, and five community-dwelling older adults), which involved a sensory rating using a validated 7-point scale meal assessment tool and a semi-structured focus group discussion. Analysis was conducted using quantitative and qualitative approaches. Soft & Bite-Sized meals had significantly higher palatability ratings than others. Sous-vide meals were most suitable for Soft & Bite-Sized texture, while commercially packaged samples were most appropriate for minced moist and pureed meals. Three main themes emerged through content analysis: (1) palatability of TMFs, (2) perceived challenges with the currently available TMFs and (3) key differences in opinion between stakeholders. Freshly made TMFs were more appealing and tastier, whereas readily prepared (pre-cooked, packaged and require reheating) TMFs had a more consistent texture. The texture of all TMFs requires enhancement, particularly in pureed meals. Developing nutritious and safe TMFs for people with dysphagia requires the promotion of active insight exchange between dietitians and speech-language therapists.

## 1. Introduction

Dysphagia (swallowing difficulties) is becoming more prevalent with the aging population, with an estimated diagnosis in 8% of the global population [1]. Patients with neurological disorders, such as stroke, brain injury, Parkinson’s disease and dementia, are more likely to experience dysphagia [2]. Swallowing difficulties can have a significant impact on mealtime safety and enjoyment. In a recent New Zealand study, older age and dysphagia are also associated with malnutrition, with up to 7% of community-dwelling adults over 65 yrs old at risk of malnutrition [3]. In order to improve swallowing efficiency and prevent choking, texture-modified foods (TMFs) are commonly prescribed to older adults with dysphagia by speech-language therapists and dietitians [4,5]. Traditionally, TMFs are modified to softer textures and smaller particle sizes with cohesive consistency through chopping, mincing, or blending with liquid and with avoidance of sticky textures, mixed consistencies and fibrous foods [6,7]. Classification of TMFs has recently been standardised by the International Dysphagia Diet Standardisation Initiative (IDDSI), which categorises TMFs into five levels: level 7—easy to chew/regular; level 6—Soft & Bite-Sized; level 5—Minced & Moist; level 4—pureed; and level 3—liquidised [8].

In recent years, the popularity of commercial TMF products has grown because of the advancement of technologies and increased demand [9]. Several companies specialise in producing TMFs for individuals with dysphagia [9]. Our previous work explored the currently available TMFs in New Zealand [10]. Auckland’s aged care facilities and hospitals either cooked their own TMFs in the kitchen, used a foodservice company that cooked off-site, or purchased commercially packaged TMF ready-meals to heat up in the kitchen [10]. Currently, commercially packaged TMF ready-meals are only available in Minced & Moist and pureed textures, with no commercially available options for Soft & Bite-Sized level. We found two other available types of TMF that have not yet been distributed in the aged care foodservice—a sous-vide meat product and a hydrolysed meat product. The sous-vide meat was cut into small pieces and cooked in a sealed bag using a water bath, which provides a soft and juicy texture. The hydrolysed meat was prepared using raw beef or chicken with a kiwifruit enzyme to produce a mild hydrolysis reaction in order to tenderise the meat proteins [11].

Mealtimes play an essential role in health outcomes and quality of life. Texture, appearance, taste and acceptance are critical factors in older people’s food consumption, while nutrition content is linked to muscle maintenance, immune function, healing and recovery [12,13,14,15,16,17]. The evidence shows that texture-appropriate and palatable TMFs can significantly improve older adults’ nutrition intake and quality of life [18]. Although TMFs are well-established, evidence suggests there are still challenges in producing palatable and standardised TMFs with optimal nutrition content [9,19,20]. A recent local study interviewed aged care residents receiving commercially packaged TMFs. The majority of consumers were satisfied overall with the TMFs [21]. However, findings were limited by resident’s communication abilities, and concerns were raised about not knowing ‘what’ they were eating and the presentation, colour and smell being less appealing. It is common for older adults suffering from dysphagia to have concurrent cognitive impairment and communication difficulties secondary to the neurological cause of their dysphagia [22]. Furthermore, this challenge perhaps accounts for the lack of consumer satisfaction research for TMFs, including among people with dysphagia [23,24,25]. In response to this, we aimed to explore liking of and attitudes towards the currently available TMFs and gain insights into the perceived challenges from dietitians, speech-language therapists and older adults. We carried out consumer testing in our study in order to gain an in-depth understanding of product acceptability and appropriateness from stakeholders—healthy older adults and health professionals involved in dysphagia care. Dietitians and speech-language therapists are the two key health professionals working with TMFs, and the healthy older adult group was chosen as surrogate consumers to understand perspectives towards TMFs. We compared four available TMFs in New Zealand: freshly made TMFs and three types of readily prepared TMFs (commercially packaged, sous-vide and hydrolysed TMFs). We evaluated liking, acceptance, expectations and opinions regarding the four meal types.

## 2. Methods

### 2.1. Study Design

This study used a mixed-methods research design which combines elements of both qualitative and quantitative approaches. The participants were recruited and divided into five focus groups to attend food test sessions. Each participant received a self-administrated anonymous questionnaire and eight TMF samples during the sessions. Participants were given 15–20 min to independently test the samples and complete the questionnaires (without talking to other participants). After completing the questionnaires, a 40–45-min focus group discussion was held. A focus group approach was adopted to enable participant interaction and stimulate in-depth discussions in order to characterise the phenomenon [26]. The Good Reporting of A Mixed Methods Study (GRAMMS) Checklist was used to guide study design and reporting [27]. This study was approved by The (removed for blind peer review) on 10 December 2019 and was conducted in September 2020 at The ([removed for blind peer review) Clinical Research Centre. All participants provided written consent.

### 2.2. Participants

Study recruitment was conducted through convenience sampling. The study was advertised in the (removed for blind peer review) hospital and university dietetic and speech-language therapists’ networks. Flyers aimed at recruiting community older adults were distributed in local community clinics and aged care facilities. Eligible dietitians and speech-language therapists needed previous experience with TMFs. Eligible older adults needed to be 65 years or older. All participants were required to be fluent in English, able to consume chicken and beef products, and with no known food allergies. Recruitment and eligibility screening were completed individually, but there were no strict limitations on whether participants were known to each other. Participants received a $20 petrol voucher to reimburse their travel costs.

### 2.3. TMF Samples

We selected two typical meals served by aged-care foodservice organisations for testing that were also available from the chosen commercial company: beef Bolognese (beef goulash for Soft & Bite-Sized level) and butter chicken. The commercially packaged meals were purchased from the supplier. Our research team prepared freshly made meals. The local hydrolysed meat supplier developed the Minced & Moist and pureed meals in their commercial kitchen following IDDSI standards and also prepared the sous-vide meals to match the Soft & Bite-Sized category. Each focus group was randomly assigned to test one of these two meal types—beef or chicken. We prepared eight samples for each type of meal: (1) freshly made Soft & Bite-Sized; (2) sous-vide Soft & Bite-Sized (raw meat was cut into 14 mm pieces and assembled with other ingredients in a vacuum-sealing bag, then slow-cooked to a precise temperature in a water bath); (3) freshly made Minced & Moist; (4) hydrolysed Minced & Moist; (5) commercially packaged Minced & Moist; (6) freshly made pureed; (7) hydrolysed pureed; (8) commercially packaged pureed. Sous-vide, hydrolysed and commercially packaged samples were provided by the local suppliers in bulk. The samples were all prepared in accordance with IDDSI specifications and tested for compliance. To reduce flavour differences across samples and replicate the actual meals served by foodservice, we used recipes provided by an aged-care foodservice organisation. All meals were made with the same primary ingredients, but in different proportions, as listed in Table 1. Nutritional content was calculated using FoodWorks (V10, Xyris Ltd., Brisbane, Australia) for freshly made, sous-vide and hydrolysed samples and based on the nutrition information panel for commercially packaged samples (Table 2).

The researchers prepared testing samples using a standard scoop (50 g ± 10 g), and participants were blinded to meal type to minimise bias. All samples were heated in an 800-Watt microwave oven for 6 min (4 min first, then rested, stirred and microwaved for another 2 min). The samples were served to participants within 10 min with a temperature of at least 65 °C. Each participant received eight randomly numbered samples (1–8). Eight samples were presented in identical plastic disposable bowls at the same time, and participants could test the samples in any order according to their preferences. A spoon and fork were provided.

### 2.4. Data Collection

#### 2.4.1. Questionnaires with Sensory Ratings

There were three sections included in the questionnaire. The first section asked for participant demographic information, including gender, age, occupation, years of experience (for dietitians and speech-language therapists only) and health conditions. The second section consisted of seven sensory evaluation questions for each sample. The questions included in the questionnaire were adapted from a validated meal assessment tool (MAT) designed to test meal components [28]. Participants were asked to rate each sample’s appearance, flavour and taste, smell, texture, and overall perceived quality on a 7-point scale (1 = extremely poor; 4 = average; 7 = excellent). Two additional questions were designed to measure participant expectations and inquire whether the sample could be easily chewed and swallowed (for older adults) or whether the sample would be appropriate for consumers requiring TMF (for dietitians and speech-language therapists). The last section asked the dietitians and speech-language therapists to choose the most suitable sample for each level. We asked the participants to rate their most important aspects of TMFs from a choice of appearance, cost, the complexity of preparation, flavour and taste, nutrition content, smell, texture and consistency, and varieties. Participants were asked to complete the sensory evaluation independently and were not able to change their answers after the focus group discussion. Based on previous study, a number of 30 participants enable the statistical differences in sensory tasting of texture-modified foods [29].

#### 2.4.2. Focus Groups

Following Onwuegbuzie et al.’s focus group framework, we developed a semi-structured topic guide that was used in all focus groups (Supplementary File S1) [30]. The guide consisted of three areas: (1) overall understanding of TMFs; (2) discussion of the samples; (3) future improvements and expectations. We asked open-ended questions to encourage further discussion and avoid providing opinions or guiding participants [26]. Focus groups were stopped when no one had anything further to add. More focus groups were initiated until the researchers agreed that data saturation had been achieved. Based on the framework, recruitment of six focus groups (three groups tested beef products and the other three groups tested chicken products) is able to achieve data saturation [30]. Each focus group included one type of stakeholder (dietitians, speech-language therapists or older adults) [31]. Using homogenous groups who had similar background, experience, education and knowledge can stimulate participant interactions and group dynamics, and make participants feel more confident to voice their views [32].

The focus groups were conducted by the same facilitator (first author, a registered dietitian), who has expertise in foodservice and TMFs. The session began with an introduction and the purpose of the study. The facilitator explained how to complete the questionnaire and commenced the meal testing. After everyone had completed the testing and questionnaires, the facilitator posed a few contextual questions: ‘Why are you interested in TMFs and have you tried it before’? ‘What was your expectation of TMFs’? This was intended to gain an understanding of the participant’s background and familiarise the participants with the topic. The focus group topic guide was then followed in the session to initiate discussion on the samples. Approximately 45 to 60 min were spent in each session.

### 2.5. Data Analysis

Stakeholder scores and other continuous data underwent descriptive analysis was performed expressed as mean and standard deviation (*SD*) for normally distributed data, while ordinal variables (7-point Likert scale) expressed as median with interquartile range [IQR, 25–75th percentile]. Categorical variables were tallied and presented as percentages and Chi-Square was performed comparing between stakeholder groups. A Kruskal-Wallis test was performed to compare the rating differences among stakeholder groups and meal types. *p*-value < 0.05 was considered as statistically significant. All statistical analyses were performed with GraphPad Prism v9.0 (GraphPad Software, Inc., San Diego, CA, USA).

The stakeholder focus group data where collected as digital recordings collected were transcribed by the facilitator (first author) who was already familiar with the data. The second author validated the accuracy of the transcription by cross-checking against the audio recordings. Transcriptions were analysed using constant comparison analysis in NVivo 12 (QSR International, Melbourne, Australia). This analysis approach was chosen to assess the consistent and distinct themes arising from different focus groups [30]. Content analysis was conducted over three stages [33]. Firstly, the data were coded into keywords, short phrases and descriptors. Then, the coded data were grouped into categories. Lastly, we emerged the main themes and subthemes from the categories. The second author reviewed the coding independently and discussed the potential categories and themes with the first author. Final themes were established after all authors reached consensus. Illustrative quotes were used to ensure participants’ voice was heard. In order to enhance rigour, the study applied triangulation by using different data collection methods (questionnaires and focus groups) and diverse data sources (three different stakeholder groups) [34].

## 3. Results

### 3.1. Participant Characteristics

Four consenting participants were unable to come to the testing sessions due to personal commitments. We could only conduct one focus group of older adults due to COVID-19 lockdowns. In total, five focus groups were conducted and each group included either four or five participants, including a total of nine dietitians, seven speech-language therapists, and five older adults (Table 3). Dietitians and speech-language therapists described a range of areas of clinical practice (aged care, head and neck cancer, oncology and stroke patients). Older adults lived in independent retirement villages or their own homes. None of the participants reported having chewing or swallowing difficulties, or any health conditions that might affect their taste. Only one participant from the older adults group reported having a loss of smell and therefore the questions about smell were not answered.

**Figure 1:**
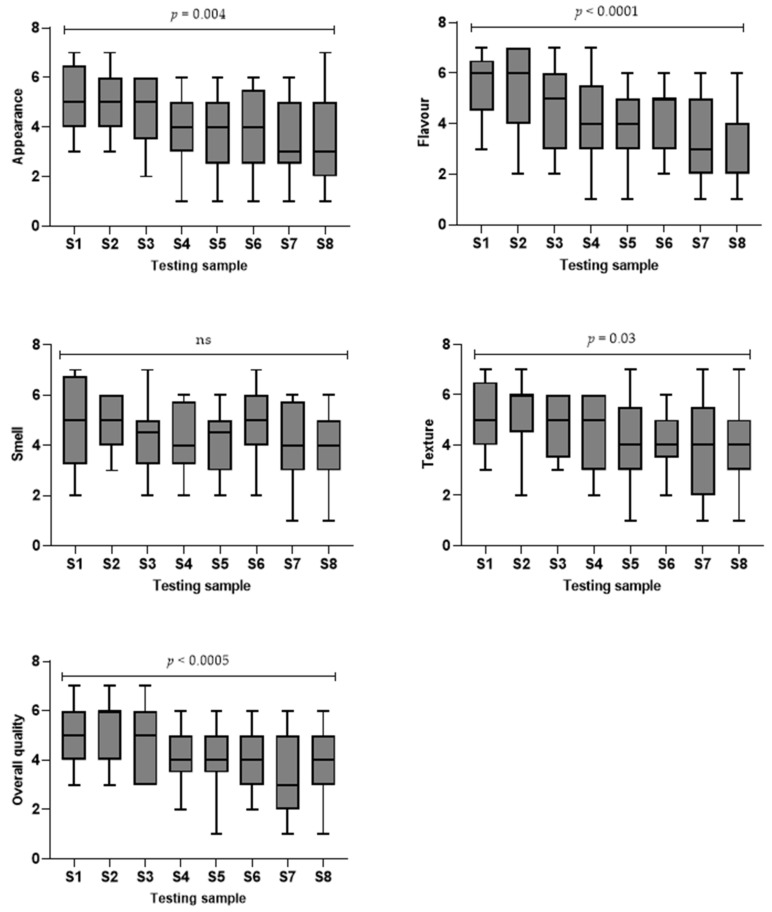
Sensory aspects of texture-modified meal testing samples using a 7-point scale. 1 = extremely poor, 4 = average, 7 = excellent. S1 = freshly made Soft & Bite-Sized sample, S2 = sous-vide Soft & Bite-Sized sample, S3 = freshly made Minced & Moist sample, S4 = hydrolysed Minced & Moist sample, S5 = commercially packaged Minced & Moist sample, S6 = freshly made pureed sample, S7 = hydrolysed pureed sample and S8 = commercially packaged pureed sample. Kruskal-Wallis test was performed. ns = no significance. *p* < 0.05 indicates significance.

Overall, Soft & Bite-Sized meals scored higher in all sensory aspects (Figure 1). Three main themes and seven subthemes have emerged, representing the key insights of TMFs from the stakeholders: (1) palatability of TMFs; (2) perceived challenges with the currently available TMFs; (3) key differences in opinion between stakeholder groups.

### 3.2. Palatability of TMFs

#### 3.2.1. Appearance Is the First Impression

In relation to the palatability of TMFs, we found three sub-themes: (1) appearance is the first impression; (2) richer meat taste is preferred; and (3) optimal texture and consistency are difficult to achieve. Appearance attributes of hydrolysed and commercially packaged pureed meals received the lowest ratings, which were below “Average”. Besides the smell, significant differences in sensory ratings were found across all samples (appearance: *p* = 0.004; flavour: *p* < 0.0001; texture: *p* = 0.03; overall quality: *p* < 0.0005). However, no significant differences were found when comparing the different meals within each level of TMF. Chi-square analysis indicated that participants who believed the samples met their expectations did not differ by meal type. All stakeholders agreed that the appearance of the samples could be off-putting, especially the colour. Freshly made, sous-vide and hydrolysed TMFs were found to have a more natural colour compared to the bright colour in commercially packaged ones. Participants also indicated that knowing the ingredients was necessary, and the colour could influence their perceptions of the ingredients.

- “I personally felt like the colour was all right because it still looks like meat. I feel like people wouldn’t want artificial colouring.” (Speech-language therapist).
- “If you can see what is in it, you kind of know what to expect. I don’t know if it is just my mind, (I feel) like all the orange ones taste the same, and then all the other colour ones taste the same.” (Dietitian).

#### 3.2.2. Richer Meat Taste Is Preferred

Despite participants’ differing preferences in flavours, a stronger meaty taste was preferred. Sous-vide and hydrolysed TMFs were considered to be the meatiest. Commercially packaged ones were described as creamy and mellow.

- “I think taste that is quite a personal thing because you can have a couple of carbs and a veg on your plate, and this is only part of your meal.” (Speech-language therapist).
- “I think (hydrolysed puree) seven tastes meatier to me compared to (commercially packaged puree) eight. (Commercially packaged puree) eight is creamier.” (Dietitian).

#### 3.2.3. Optimal Texture and Consistency Are Difficult to Achieve

There was mixed opinion about texture and consistency. Significant differences were found in the appropriateness question, where freshly made Soft & Bite-Sized meals were considered less appropriate than others, X^2^ (7, *n* = 21) = 16.4, *p* = 0.02. Sous-vide meals were chosen as the most suitable Soft & Bite-Sized texture (*n* = 7, 44%), while commercially packaged were rated as the most suitable Minced & Moist (*n* = 7, 44%) and pureed texture (*n* = 9, 56%). Several participants found that freshly made TMFs were too watery, while others indicated that pureed ones could be gritty and dry out over time. All stakeholders agreed that a moist and smooth texture is easier to swallow.

Multiple participants indicated that the meat in freshly made Soft & Bite-Sized samples was too tough. Despite the sous-vide samples being selected as the most suitable Soft & Bite-Sized meal, speech-language therapists were dissatisfied with inconsistent meat chunk sizes and texture. Dietitians suggested that different cuts of meat can affect the tenderness. For example, lean chicken breasts can be chewy due to their high muscle fibre content, while thighs with more fat would be easier to chew.

- “I thought waterier was nicer. This one (commercially packaged Minced & Moist) was a bit dry. I mean it is probably moister than it looked, which is why I did call it meeting standard.” (Speech-language therapist).
- “I didn’t find six (freshly made puree) that acceptable as a smooth puree. I do find it quite gritty and for my head and neck patients who may have quite a lot of swelling and when they’ve reconstructed. Say somebody’s tongue doesn’t move the same as your tongue does and things get lost down the side of the mouth, so that sort of grittiness that people have commented on quite a lot not with the puree food.” (Dietitian).
- “I didn’t like the first one (freshly made Soft & Bite-sized chicken). I found it was a bit too chewy and tough. But the second one (sous-vide Soft & Bite-sized chicken), those smaller chicken pieces, were divine.” (Older adult).
- “The (sous-vide Soft & Bite-sized) two is closer (to the standard), but still need to really focus on the chewing and put some force into it. I just wonder if beef and lamb are virtually impossible to make Soft & Bite-Sized as the meat product.” (Speech-language therapist).

### 3.3. Perceived Challenges for the Currently Available TMFs

#### 3.3.1. Freshly Made TMFs

In relation to perceived challenges for the currently available TMFs, we summarised the subthemes based on (1) freshly made TMFs and (2) readily made TMFs. Freshly made samples were commented as having better flavour but inconsistent in texture. When using a blender, it can be difficult to process freshly made TMF into the standardised levels. Both stakeholders believed that education is necessary to guide foodservice staff and caregivers in preparing appropriate TMFs.

- “In terms of caregiver, I don’t think there is enough training for caregivers to prepare the same consistency (TMFs).” (Speech-language therapist).
- “We have two soft diets. We have got soft mechanical and soft dysphagic, so sometimes there can be confusion around which one is most appropriate. It is really important to differentiate like what do you consider soft does.” (Dietitian).
- “From the foodservice perspective, I am just thinking about whenever I have been in there. It is almost like the foodservice staff will identify which sort of food options are in the standard menu that would qualify as Soft & Bite-Sized.” (Dietitian).

#### 3.3.2. Readily Prepared TMFs

Dietitians often assist patients in choosing appropriate foods based on their prescribed level of TMF. Dietitians pointed out that preparing TMFs can be challenging for older patients, particularly when fortification is required. Therefore, commercially packaged TMFs were commonly recommended to older patients after discharge.

Both hydrolysed and commercially packaged TMFs were more consistent in texture and met the Minced & Moist and pureed standards. However, commercially packaged TMFs lacked variety, in particular cultural flavour. Despite readily available TMFs being time-saving, preparation and serving can sometimes be inappropriate.

- “It depends on their age. I mean, obviously that we do get a lot of young patients around 30–50 s that are perfectly capable of preparing food, and then we get a lot of older patients who may not necessarily be that comfortable with doing that or don’t have the energy to do that. So, we can give them advice for home cooking and talk about food fortification of modified textured foods. We quite often point them towards the commercially packaged ones.” (Dietitian).
- “There is not much variety, so I think it will be good to have some varieties with different things and when it is ready-made as well.” (Speech-language therapist).
- “Because I know the hospital uses the products from the commercially packaged company. I think there is a difference between somebody using those products and warming them up themselves at home and then being done on an industrial scale and delivered to the patients. They are not necessarily arriving at the patient’s bedside in the most appropriate way.” (Dietitian).

### 3.4. Key Differences in Opinions between Stakeholder Groups

#### 3.4.1. Different Choices in Preferred TMFs

Two subthemes were responsible for the main differences in opinion between groups: (1) different choices in preferred TMFs and (2) different focus of TMF between dietitians and speech-language therapists. The median sensory test scores of the three stakeholder groups were compared with respect to specific attributes from each sample (Supplementary File S2). The most noteworthy variance was found in the freshly made Soft & Bite-Sized sample, where speech-language therapists had significantly lower acceptance of the appearance (*p* < 0.0001), flavour (*p* = 0.001) and smell (*p* = 0.01) than dietitians and older adults.

Other significant differences between groups were found in the perceived appearance of sous-vide (*p* = 0.003) and freshly made Minced & Moist meals (*p* = 0.03), the texture of hydrolysed Minced & Moist (*p* = 0.03) and pureed meals (*p* = 0.01), and the overall quality rating of commercially packaged Minced & Moist (*p* = 0.005) and hydrolysed pureed meals (*p* = 0.02). The hydrolysed and commercially packaged meals were not well received by the older adults, as evidenced by the lower ratings compared to those of dietitians and speech-language therapists. Older adults found that Minced & Moist and pureed meals were bland, and it was hard to distinguish the flavours.

**Figure 2:**
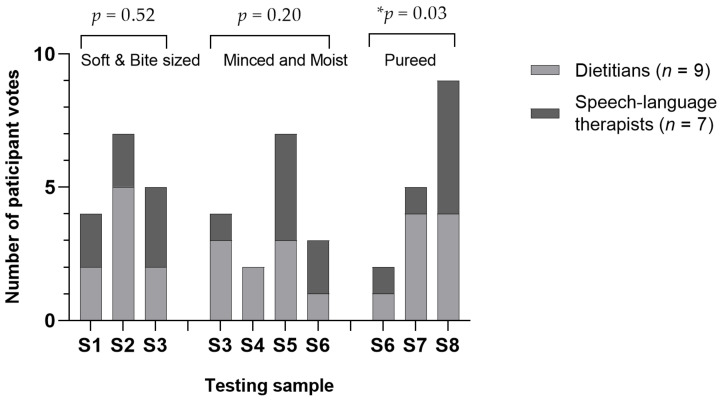
Healthcare professional votes for each level of texture-modified foods (*n* = 16). S1 = freshly made Soft & Bite-Sized sample, S2 = sous-vide Soft & Bite-Sized sample, S3 = freshly made Minced & Moist sample, S4 = hydrolysed Minced & Moist sample, S5 = commercially packaged Minced & Moist sample, S6 = freshly made pureed sample, S7 = hydrolysed pureed sample and S8 = commercially packaged pureed sample. Chi-square test was performed to test the categorical variables. * *p* < 0.05 indicates significance.

There were no significant differences between dietitians and speech-language therapists in selecting the samples most appropriate to each level of TMFs (Soft & Bite-Sized *p* = 0.53, Minced & Moist *p* = 0.35 and pureed *p* = 0.43). Overall, commercially packaged pureed samples received significantly more votes than other pureed samples (Figure 2).

- “I feel like five (commercially packaged minced & moist) and eight (commercially packaged puree) was what I expected like texture-modified food to taste like, just quite tasteless and like not very pleasant. The last one (commercially packaged puree) tastes like pumpkin, but I was surprised by the other ones because it was better than I expected.” (Older adult).

#### 3.4.2. Different Focus of TMF between Dietitians and Speech-Language Therapists

**Figure 3:**
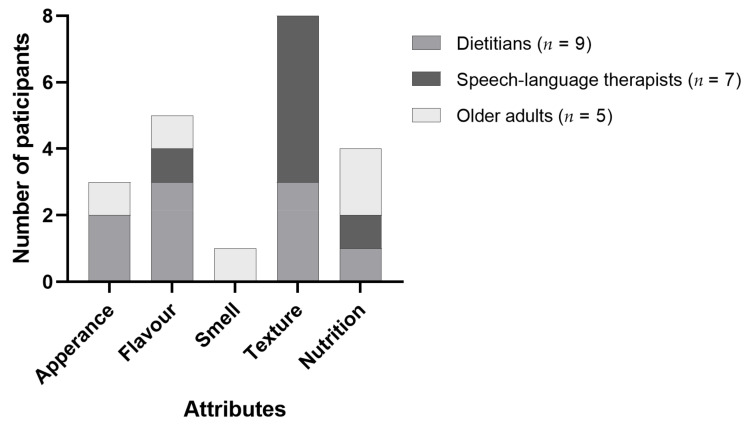
The most important attributes of texture-modified foods ranked by participants from focus groups (*n* = 21).

As shown in Figure 3, 71% (*n* = 15) of the participants chose texture as the top attribute, followed by flavour (67%, *n* = 14) and nutrition (62%, *n* = 13). Dietitians and speech-language therapists had varying perspectives on an ideal TMF. Dietitians focused on patient intake, which is associated with the palatability and nutrition content of TMFs, whilst speech-language therapists placed a higher priority on texture and consistency, which in turn affects patient safety. A higher protein level and meat component were attractive to all stakeholders.

- “I feel flavour and taste (are the most important things) because I think texture makes sense logically, but I feel like you can’t really have one without the other. With flavour, like when I go to see patients in the hospital, the main reason people weren’t eating enough when they were on texture-modified foods was because they just didn’t like the taste. They just found it was disgusting and no texture, appearance, smell or anything could overcome that. So, I feel like flavour is really important because no matter how the texture is if they are not going to eat it.” (Dietitian).
- “I guess our job as a speech-language therapist is always about that safety, so from a speech therapist’s point of view, we care about lumps because they are safety risks for choking.” (Speech-language therapist).
- “I would definitely go for the one with higher meat content.” (Older adult).

## 4. Discussion

This study aimed to explore key stakeholders’ perceptions of currently available TMFs through a validated meal assessment rating tool and targeted focus groups. To our knowledge, this is the first study to evaluate three IDDSI levels of TMFs using analytical sensory rating and in-depth insights from stakeholders. The study focused on identifying the appropriateness and challenges of the TMFs, providing a basis for future TMF development. Soft & Bite-Sized samples were more acceptable to participants than Minced & Moist and pureed samples. Selected samples were made from matched ingredients but with different processing techniques. There were wide differences in meat to non-meat ratios, nutritional content and stakeholder feedback on texture. Stakeholders preferred a meatier flavour. Yet, while sensory ratings varied significantly across samples, no significant difference was found across different processing techniques within the same TMF level.

### 4.1. Palatability of TMFs

Appearance and flavour were both strong determinants of satisfaction with TMFs, particularly in dietitians and older adults. These results are in line with previous studies evaluating TMF satisfaction of older adults with dysphagia [19,23,29,35]. Our results indicate that the samples with lower appearance ratings were less likely to meet participants’ expectations. Ettinger et al. found that appearance was positively associated with flavour and texture liking among TMF consumers [23]. Therefore, visually appealing TMFs have a positive effect on nutrition intake [20]. Freshly made samples were the most palatable type of TMFs, yet dietitians and speech-language therapists voiced concerns about texture and consistency. Lack of standardised production is a challenge in freshly made TMFs, with the absence of standardised recipes, processing techniques and lack of education [10,19,36]. Previous studies demonstrate success in improving freshly made TMFs by implementing staff training and providing cooking workshops to patients and caregivers [37,38]. In contrast to freshly made TMFs, readily prepared TMFs offer the advantage of consistent mass production and reliable texture, but are limited by personal preferences and food varieties [19]. Miles et al. reported that only half of aged care consumers were satisfied with the variety of commercially packaged meals [21].

Commercial packaged TMFs, sous-vide and hydrolysed meats can be prepared in bulk by the food industry and packaged into small servings and stored in the freezer. Hydrolysed TMFs offer flexibility in customising meal flavours, colours and presentations [39]. Hydrolysed meat can be packaged without flavour and serve as a protein in any meal. By adding different garnishes, spices, herbs and sauces to hydrolysed meat, consumers can modify the flavour easily without suffering sensory fatigue.

### 4.2. Nutritional Content of TMFs

Palatability is influenced by fat and liquid content, processing techniques and thickening agents [4,19,29,40]. Processing techniques within the same TMF did not significantly impact the sensory ratings, but all stakeholders preferred samples with a meatier taste, indicating that consumers place a high priority on the taste of the key ingredient. Based on nutritional content analysis, hydrolysed meat contains the highest protein content with the least saturated fat and carbohydrates, making it ideal for vulnerable older adults. Similar to other commercial TMFs studied, the readily prepared TMFs in our study are enriched with protein either through processing technology or protein fortification [19]. However, the meat content varied greatly based on processing technique, with a maximum difference of 35% between commercially packaged (31%) and hydrolysed samples (66%) of butter chicken meals. Meat fortification with functional proteins requires careful selection due to the variable effects on texture characteristics and nutritional content [41]. The addition of pea protein to commercially packaged TMFs softens the texture and enriches the protein content, but the taste was artificial or diluted. This finding supports previous research into commercial thickeners, which enhanced the appearance but had lower acceptance regarding taste and overall liking [40]. Compared to the readily prepared meals, the freshly made TMFs had the least protein content and the highest sodium content in all levels. Freshly made TMFs are often challenged by the fact that their nutrition content is diluted during processing, and therefore require a larger volume to achieve optimal nutrition [19,42].

In spite of inconclusive evidence regarding the optimal sensory characteristics of TMFs, freshly made TMFs that are closer to homemade dishes may improve consumption in older adults [37]. When developing TMFs, the flavour should perhaps be as close to the original meals as possible. Hydrolysed meat and sous-vide cooking techniques are promising technologies used for meat tenderisation without diluting the nutrition content and meat flavour. However, other techniques may be needed to ensure visual palatability, such as three-dimensional (3D) food printing which has been used to create different textured foods with enhanced sensory characteristics and reduced fabrication time [43]. A recent review suggests that the combined application of non-thermal technologies (high-pressure processing) and 3D printing can be a potential quality improvement for patients with dysphagia [44].

This study was unable to measure the actual cost of each type of production. However, none of the stakeholders rated “cost” as their prior consideration when it comes to the choice of TMFs. While commercial products can be considered more expensive, some researchers have postulated that the freshly made TMFs may be more costly due to the negative implications of inappropriate meal texture and nutrient levels leading to risks of choking and aspiration, food wastage and inadequate nutrition density [36]. Further study should consider including a financial evaluation that takes into account the cost of products/ingredients, labour preparation, the potential cost of hospitalisation, and medical complications, such as malnutrition, choking and aspirations.

### 4.3. Differences between Stakeholders

Consistent with other sensory evaluation studies, this research found that each stakeholder group had different attitudes towards TMFs [23,40]. Interestingly, while speech-language therapists ranked patient safety and texture highly, dietitians’ first choice was appearance, arguing that, if the meal was unappealing, patients would refuse to eat it, leading to malnutrition and other health complications. This is likely to be explained by the differing responsibilities of health professionals, with speech-language therapists assessing swallow safety and efficiency, and dietitians attending to overall nutrition intake [45,46]. Dietitians and speech-language therapists working together and performing joint evaluations undoubtedly improves patient-centered care through these multiple lenses [46,47]. Our study assessed the health professionals in different groups based on their professions. Future studies may consider having inter-professional joint focus groups to understand the different viewpoints and opportunities for partnership in developing appropriate TMFs.

### 4.4. Limitations

TMFs are primarily consumed by individuals who have difficulty chewing or swallowing. However, communication comorbidities are common in those with dysphagia making it difficult to conduct in-depth verbal discussions and objective ratings [21,36]. The health professionals and older adults in this study are not truly representative of consumers suffering from dysphagia but have expertise or experience with swallowing or chewing difficulties. Older adults with dysphagia have been shown to have different preferences and judgments of palatability to their health professionals [23,48]. Participants without impaired chewing and/or swallowing abilities and prior exposure to pureed products possibly have lower acceptance levels [23,49]. Irrespective, our stakeholders provided valuable insights. We had a heterogeneity group containing dietitians and speech-language therapists due to the recruitment difficulty. Despite the different professions, participants had similar experience in patients with dysphagia and knowledge of texture-modified diets. It is also known that dietitians and speech-language therapists often work together and the moderator also had extensive experience working with both professionals, therefore, the heterogeneity influence was limited.

Variations in ingredients between samples may have influenced sensory testing. However, we aimed to compare the currently available products and maintain their sensory characteristics without alternations. Variation in sample sizes across the three stakeholder groups due to the COVID-19 restrictions for vulnerable groups may have affected the findings [50]. Future research should involve larger sample sizes, including people with dysphagia, foodservice staff and food scientists.

## 5. Conclusions

In conclusion, readily prepared TMFs are promising food products that offer a consistent texture and ease of preparation. However, the appearance and flavour of Minced & Moist and pureed levels of TMFs still require improvement. For people with dysphagia who prefer freshly made TMFs, preparation standards (such as texture, consistency and particle sizes) and patient safety should be considered. Hydrolysed meat products hold promise in offering a protein-dense and IDDSI-compliant product when incorporated into freshly made meals. Dietitians and speech-language therapists have different roles, and both need to share their insights and expertise in order to assist patients and foodservice establishments in selecting the most suitable TMFs.

## Figures and Tables

**Table 1:** Ingredient composition in sample texture-modified meals used in the focus group.

	Beef Bolognese (Beef Goulash for Soft & Bite-Sized Level)		
	Freshly Made	Sous-Vide/Hydrolysed	Commercially Packaged
Beef (%)	38	60–75	61
Tomato puree (%)	30	10	12
Vegetables (%)	26	2–17	10
Others ^a^ (%)	6	13	17
	Butter chicken		
Chicken (%)	47	66	31
Cream (%)	12	9	19
Tomato puree (%)	19	2	14
Onions (%)	10	3	36 ^b^
Others ^a^ (%)	12	20	

^a^ Others include oil, salt, spice, stock and water. Hydrolysed meals contain kiwifruit enzyme and commercially packaged meals are fortified with pea protein and citrus fiber. ^b^ The proportion of onions was unspecified, therefore 36% includes onions, sugar, Greek yogurt and others as listed above.

**Table 2:** The nutritional content of freshly made and readily prepared texture-modified meals used in the focus group (per 100 g).

Product	Levels	Type	Energy (kJ)	Protein (g)	Fat (g)	Saturated Fat (g)	Carbohydrate (g)	Dietary Fibre (g)	Sodium (g)
Beef Bolognese	Soft & Bite-Sized	Freshly made	387	10.1	4.3	1.2	3	0.7	742
		Sous-vide	676	16.5	7.2	1.4	5.7	4.3	448
	Minced & Moist	Freshly made	396	6	6.2	3	2.7	0.9	678
		Hydrolysed	498	14.2	5.6	2.4	2.5	1.4	255
		Commercial	723	17	9.4	2.9	5.7	1.1	370
	Pureed	Freshly made	396	6	6.2	3	2.7	0.9	678
		Hydrolysed	599	17.7	7	3	2	1.1	423
		Commercial	723	17	9.4	2.9	5.7	1.1	370
Butter chicken	Soft & Bite-Sized	Freshly made	400	10.6	4.4	2.5	2.9	1	794
		Sous-vide	375	15.5	1.7	0.6	2.7	1	287
	Minced & Moist	Freshly made	400	10.6	4.4	2.5	2.9	1	794
		Hydrolysed	375	15.5	1.7	0.6	2.7	0.9	288
		Commercial	738	11.2	11.3	5.8	7.6	1.9	216
	Pureed	Freshly made	400	10.6	4.4	2.5	2.9	1	794
		Hydrolysed	374	15.5	1.7	0.6	2.7	0.9	288
		Commercial	738	11.2	11.3	5.8	7.6	1.9	216

**Table 3:** Participant characteristics from the focus groups.

	Dietitians (*n =* 9)	Speech-Language Therapists (*n* = 7)	Older Adults (*n =* 5)	Total (*n =* 21)
Gender (Male/Female)	0/9	0/7	1/4	1/20
Mean age (years) ± SD	29 ± 11	29 ± 8	72 ± 4	39 ± 21
Age range (years)	20–50	24–45	68–77	20–77
Professional area ^a^				
Hospital	4	1	Retired	5
Research	2	1		3
Master students ^b^	4	5		9
Commercial industry	1	0		1

^a^ Participants may work in 2 professional areas (1 dietitian works in hospital/research, 1 dietitian works in hospital/commercial industry); ^b^ All student participants were in their last year of their Master’s degree and have completed clinical placements.

## Data Availability

The datasets used and/or analysed during the current study are available from the corresponding author on reasonable request.

## Supplementary Materials

The following supporting information can be downloaded at: https://www.mdpi.com/article/10.3390/foods11142157/s1, Supplementary File S1: Focus group schedule guide; Supplementary File S2: Stakeholder comparisons.

## References

[1] Cichero J.A.Y., Steele C., Duivestein J., Clavé P., Chen J., Kayashita J., Dantas R., Lecko C., Speyer R., Lam P. (2013). The Need for International Terminology and Definitions for Texture-Modified Foods and Thickened Liquids Used in Dysphagia Management: Foundations of a Global Initiative. Curr. Phys. Med. Rehabil. Rep..

[2] DeFabrizio M.E., Rajappa A. (2010). Contemporary Approaches to Dysphagia Management. J. Nurse Pract..

[3] Wham C., Fraser E., Buhs-Catterall J., Watkin R., Gammon C., Allen J. (2017). Malnutrition risk of older people across district health board community, hospital and residential care settings in New Zealand. Australas. J. Ageing.

[4] Rothenberg E., Wendin K., Chen J., Rosenthal A. (2015). Texture modification of food for elderly people. Modifying Food Texture.

[5] Engh M.C.N., Speyer R. (2022). Management of Dysphagia in Nursing Homes: A National Survey. Dysphagia.

[6] Cichero J.A.Y. (2016). Adjustment of Food Textural Properties for Elderly Patients. J. Texture Stud..

[7] Garcia J.M., Chambers E. (2010). Managing Dysphagia Through Diet Modifications. AJN Am. J. Nurs..

[8] Martineau C. International Dysphagia Diet Standardisation Initiative: IDDSI Framework. http://iddsi.org/framework/.

[9] Aguilera J.M., Park D.J. (2016). Texture-modified foods for the elderly: Status, technology and opportunities. Trends Food Sci. Technol..

[10] Wu X.S., Miles A., Braakhuis A. (2022). An Evaluation of Texture-Modified Diets Compliant with the International Dysphagia Diet Standardization Initiative in Aged-Care Facilities Using the Consolidated Framework for Implementation Research. Dysphagia.

[11] Ahmad N.B. (2016). Beef hydorlysis by Zyactinase Enzymes. Ph.D. Thesis.

[12] Lee K.M., Song J.-A. (2015). Factors influencing the degree of eating ability among people with dementia. J. Clin. Nurs..

[13] Cardello A.V., Bell R., Kramer F.M. (1996). Attitudes of consumers toward military and other institutional foods. Food Qual. Prefer..

[14] Cassens D., Johnson E., Keelan S. (1996). Enhancing taste, texture, appearance, and presentation of pureed food improved resident quality of life and weight status. Nutr. Rev..

[15] Mitchell C.J., Milan A.M., Mitchell S.M., Zeng N., Ramzan F., Sharma P., Knowles S.O., Roy N.C., Sjödin A., Wagner K.-H. (2017). The effects of dietary protein intake on appendicular lean mass and muscle function in elderly men: A 10-wk randomized controlled trial. Am. J. Clin. Nutr..

[16] Bauer J., Biolo G., Cederholm T., Cesari M., Cruz-Jentoft A.J., Morley J.E., Phillips S., Sieber C., Stehle P., Teta D. (2013). Evidence-Based Recommendations for Optimal Dietary Protein Intake in Older People: A Position Paper From the PROT-AGE Study Group. J. Am. Med. Dir. Assoc..

[17] Ministry of Health (2013). Food and Nutrition Guidelines for Healthy Older People A Background Paper.

[18] Zanini M., Bagnasco A., Catania G., Aleo G., Sartini M., Cristina M.L., Ripamonti S., Monacelli F., Odetti P., Sasso L. (2017). A Dedicated Nutritional Care Program (NUTRICARE) to reduce malnutrition in institutionalised dysphagic older people: A quasi-experimental study. J. Clin. Nurs..

[19] Keller H., Chambers L., Niezgoda H., Duizer L. (2012). Issues associated with the use of modified texture foods. J. Nutr. Health Aging.

[20] Wu X.S., Miles A., Braakhuis A. (2020). Nutritional Intake and Meal Composition of Patients Consuming Texture Modified Diets and Thickened Fluids: A Systematic Review and Meta-Analysis. Healthcare.

[21] Miles A., Dennison K., Oad M.A., Shasha L., Royal M. (2019). Consumer Satisfaction of Texture Modified Meals Served in Residential Aged-Care Facilities. Int. J. Food Sci. Nutr. Res..

[22] Baijens L.W.J., Clavé P., Cras P., Ekberg O., Forster A., Kolb G., Leners J.C., Masiero S., Mateos del Nozal J., Ortega O. (2016). European Society for Swallowing Disorders – European Union Geriatric Medicine Society white paper: Oropharyngeal dysphagia as a geriatric syndrome. Clin. Interv. Aging.

[23] Ettinger L., Keller H.H., Duizer L.M. (2014). A comparison of liking of pureed food between two groups of older adults. J. Nutr. Gerontol. Geriatr..

[24] Pelletier C.A., Lawless H.T. (2003). Measuring taste acceptance in neurologically impaired adults. Food Qual. Prefer..

[25] Rothenberg E., Ekman S., Bülow M., Möller K., Svantesson J., Wendin K. (2007). Texture-modified meat and carrot products for elderly people with dysphagia: Preference in relation to health and oral status. Scand. J. Food Nutr..

[26] Krueger R.A., Casey M.A., Mary A.W. (2009). Focus Groups: A Practical Guide for Applied Research.

[27] O’Cathain A., Murphy E., Nicholl J. (2008). The quality of mixed methods studies in health services research. J. Health Serv. Res. Policy.

[28] Hannan-Jones M., Capra S. (2017). Developing a valid meal assessment tool for hospital patients. Appetite.

[29] Okkels S.L., Saxosen M., Bügel S., Olsen A., Klausen T.W., Beck A.M. (2018). Acceptance of texture-modified in-between-meals among old adults with dysphagia. Clin. Nutr. ESPEN.

[30] Onwuegbuzie A.J., Dickinson W.B., Leech N.L., Zoran A.G. (2009). A Qualitative Framework for Collecting and Analyzing Data in Focus Group Research. Int. J. Qual. Methods.

[31] Wallace S., Holloway I. (2005). Observing method: Recognizing the significant of belief, discipline, position and documentation in observational studies. Qualitative Research in Health Care.

[32] Grønkjær M., Curtis T., De Crespigny C., Delmar C. (1970). Analysing group interaction in focus group research: Impact on content and the role of the moderator. Qual. Stud..

[33] Corbin J., Strauss A. (2007). Basics of Qualitative Research (3rd ed.): Techniques and Procedures for Developing Grounded Theory.

[34] Holloway I., Todres L., Holloway I. (2005). The status of method: Flexibility, consistency and coherence. Qualitative Research in Health Care.

[35] Keller H., Duizer L.M. (2014). What do consumers think of pureed food? Making the most of the indistinguishable food. J. Nutr. Gerontol. Geriatr..

[36] Ilhamto N., Anciado K., Keller H.H., Duizer L.M. (2014). In-House Pureed Food Production in Long-Term Care: Perspectives of Dietary Staff and Implications for Improvement. J. Nutr. Gerontol. Geriatr..

[37] Costa A., Carrión S., Puig-Pey M., Juárez F., Clavé P. (2019). Triple adaptation of the mediterranean diet: Design of a meal plan for older people with oropharyngeal dysphagia based on home cooking. Nutrients.

[38] Wu X.S., Miles A., Braakhuis A.J. (2022). The Effectiveness of International Dysphagia Diet Standardization Initiative—Tailored Interventions on Staff Knowledge and Texture-Modified Diet Compliance in Aged Care Facilities: A Pre-Post Study. Curr. Dev. Nutr..

[39] Anciado K., Ilhamto N., Keller H., Duizer L. (2012). Purchasing commercially prepared pureed foods: Nutrition managers’ perspectives. J. Foodserv. Manag. Educ..

[40] Stahlman L.B., Garcia J.M., Hakel M., Chambers IV E. (2000). Comparison ratings of pureed versus molded fruits: Preliminary results. Dysphagia.

[41] Baugreet S., Kerry J.P., Botineştean C., Allen P., Hamill R.M. (2016). Development of novel fortified beef patties with added functional protein ingredients for the elderly. Meat Sci..

[42] Massoulard A., Bonnabau H., Gindre-Poulvelarie L., Baptistev A., Preux P.M., Villemonteix C., Javerliat V., Fraysse J.L., Desport J.C. (2011). Analysis of the food consumption of 87 elderly nursing home residents, depending on food texture. J. Nutr. Health Aging.

[43] Sungsinchai S., Niamnuy C., Wattanapan P., Charoenchaitrakool M., Devahastin S. (2019). Texture Modification Technologies and Their Opportunities for the Production of Dysphagia Foods: A Review. Compr. Rev. Food Sci. Food Saf..

[44] Giura L., Urtasun L., Belarra A., Ansorena D., Astiasarán I. (2021). Exploring tools for designing dysphagia-friendly foods: A review. Foods.

[45] Nakamura T., Amano N. (2019). Proposal for preventing malnutrition in individuals on a texture-modified diet. Nutr. Health.

[46] Heiss C.J., Goldberg L., Dzarnoski M. (2010). Registered dietitians and speech-language pathologists: An important partnership in Dysphagia management. J. Am. Diet. Assoc..

[47] Brody R.A., Touger-Decker R., VonHagen S., O’Sullivan Maillet J. (2000). Role of registered dietitians in dysphagia screening. J. Am. Diet. Assoc..

[48] Macqueen C.E., Taubert S., Cotter D., Stevens S., Frost G.S. (2003). Which commercial thickening agent do patients prefer?. Dysphagia.

[49] Schiffman S.S., Graham B.G. (2000). Taste and smell perception affect appetite and immunity in the elderly. Eur. J. Clin. Nutr..

[50] Walton K., Williams P., Tapsell L. (2006). What do stakeholders consider the key issues affecting the quality of foodservice provision for long-stay patients?. J. Foodserv..

