# Supplementary material for: Attitudes towards Freshly Made and Readily Prepared Texture-Modified Foods among Speech-Language Therapists, Dietitians, and Community-Dwelling Older Adults
Source: Foods. 2022 Jul 20;11(14):2157. doi: 10.3390/foods11142157 (PMC9317743; doi:10.3390/foods11142157)
Supplement: Supplementary file 1 [file foods-11-02157-s001.zip › foods-1814436-supplementary-final/Supplementary File S1 - Focus group guide.pdf]

Supplementary File S1 - Focus group schedule guide

- Introduce the facilitator and the assistant.
- Hand out the participant information sheets (PIS) and consent forms (CF).
- Explain the meal tasting session, the purpose of the study and the session will be taped.
    - You were selected because we are seeking some advice and opinions from health professionals
    - There're no right or wrong answers, only differing points of view. So, we would like honest feedback from you.
    - We're tape recording for gathering the most accurate information, so please we'll try to have one person speaking at a time.
    - Before we start, I'd ask you to turn your phones into silent.
    - My role as the facilitator today will be to guide the discussion.
- After participants sign the CF, will hand out and explain how to complete the checklist and start with meal tasting (10-15min).
- The facilitator will initiate the group discussion after everyone completed the checklist.
- Topic 1: Background

    Health professionals' group

    - Why are you interested in texture-modified foods (TMFs)?
    - What do you consider the current challenges of TMFs?
    - What's your expectation of texture-modified meat (TMM)?

    Older adults' group:

    - Has anyone tried this type of meal before? If so, where did you get them or how did you prepare them?
    - What's your experience with this type of meal? Do you like it or not? Why?
- Topic 2: Tested meals
    - How do you like the food today? Were you satisfied?
    - Can you tell the main differences between these meals?
    - Are you able to identify the type of meat served?
    - What do you think of the flavour and taste?
    - How about the appearance?
    - What do you think of the texture of the food?

    Health professionals' group

    - Within samples 1 and 2, which one would you suggest the foodservice to provide for residents on a Soft & Bite-sized diet? Why? What are the problems with the meals?

    - Within samples 3-5, which one would you suggest the foodservice to provide for the residents on the Minced & Moist diet? Why? What are the problems with the meals?
    - Within samples 6-8 which one would you suggest the foodservice provide for the residents on the Pureed diet? Why? What are the problems with the meals?

    <u>Oder adults' group</u>

    - Sample 1 and 2 is soft & bite-sized meat what do you expect with soft meals? What are the problems with these meals? (e.g., texture, dryness, appearance, tastes…). Which sample do you like the most? Why?
    - Sample 3-5 is minced & moist meat, what do you expect with minced meals? What are the problems with these meals? Which sample do you like the most? Why?
    - Sample 6-8 is pureed meat, what do you expect with pureed meals? What are the problems with these meals? Which sample do you like the most? Why?

    <u>All</u>

    - Of all the things we discussed, what to you is the most important of these meals?
    - Say if any of the samples have higher protein content or are better for bowel movement, would that change your choice?
- Show the ingredient lists and cost poster
- Topic 3: Future improvements
    - What would you like to change to improve the quality of these meals?
- Have we missed anything? Is there anything else you would like to discuss about these TMM?
