# Supplementary material for: Attitudes towards Freshly Made and Readily Prepared Texture-Modified Foods among Speech-Language Therapists, Dietitians, and Community-Dwelling Older Adults
Source: Foods. 2022 Jul 20;11(14):2157. doi: 10.3390/foods11142157 (PMC9317743; doi:10.3390/foods11142157)
Supplement: Supplementary file 1 [file foods-11-02157-s001.zip › foods-1814436-supplementary-final/Supplementary File S2.pdf]

Supplementary File S2. Stakeholder comparisons

Median [IQR, 25-75th percentile] rating from the sensory test (*n* = 21)

	Soft and bite-sized		Minced and moist			Pureed		
	Freshly made	Sous Vide	Freshly made	Hydroly-sed	Commer-cial	Freshly made	Hydroly-sed	Commer-cial
**Appearance**								
Dietitians	6.0 [5.0-6.0]	4.0 [4.0-5.5]	4.0 [2.0-4.5]	4.0 [3.0-5.5]	4.0 [3.5-5.0]	3.0 [2.0-4.5]	4.0 [3.0-5.5]	3.0 [2.5-5.0]
SLTs	4.0 [3.0-4.0]	5.0 [3.0-5.0]	6.0 [5.0-6.0]	4.0 [4.0-5.0]	4.0 [3.0-5.0]	4.0 [2.0-6.0]	4.0 [2.0-6.0]	3.0 [2.0-4.0]
Older adults	7.0 [7.0-7.0]	7.0 [6.0-7.0]	5.0 [3.5-6.0]	3.0 [1.5-4.5]	2.0 [1.5-4.5]	4.0 [3.0-5.5]	2.0 [1.0-4.0]	4.0 [2.0-5.0]
P-value	**<.0001**	**.003**	**.03**	.22	.27	.47	.12	.71
**Flavour**								
Dietitians	6.0 [5.5-7.0]	4.0 [4.0-5.5]	5.0 [3.0-6.0]	4.0 [3.0-5.5]	5.0 [3.5-5.0]	5.0 [2.0-5.5]	3.0 [1.5-6.0]	3.0 [1.5-4.5]
SLTs	4.0 [3.0-5.0]	6.0 [4.0-7.0]	5.0 [5.0-6.0]	4.0 [4.0-6.0]	4.0 [3.0-5.0]	5.0 [3.0-5.0]	4.0 [3.0-5.0]	4.0 [3.0-4.0]
Older adults	6.0 [6.0-7.0]	7.0 [5.5-7.0]	4.0 [3.5-6.5]	3.0 [2.5-4.0]	3.0 [2.0-3.5]	4.0 [3.0-5.0]	2.0 [1.0-2.5]	4.0 [1.5-4.5]
P-value	**.001**	.06	.63	.12	.10	.85	.09	.67
**Smell**								
Dietitians	6.0 [4.5-7.0]	4.0 [4.0-6.0]	5.0 [4.0-5.5]	4.0 [3.5-5.5]	5.0 [3.5-6.0]	5.0 [4.0-6.0]	5.0 [3.5-6.0]	5.0 [3.5-5.0]
SLTs	3.0 [2.0-5.0]	5.0 [4.0-6.0]	4.0 [3.0-5.0]	5.0 [4.0-6.0]	5.0 [3.0-5.0]	5.0 [5.0-5.0]	4.0 [4.0-6.0]	3.0 [2.0-4.0]
Older adults	6.0 [4.3-7.0]	5.5 [3.5-6.0]	3.5 [3.0-5.5]	2.5 [2.0-5.3]	2.0 [2.0-5.0]	4.0 [2.5-5.5]	2.0 [1.0-4.5]	3.5 [1.5-4.0]
P-value	**.01**	.82	.42	.21	.26	.52	.14	.12
**Texture**								
Dietitians	6.0	5.0	6.0	5.0	5.0	5.0	5.0	4.0

	[4.0-7.0]	[4.5-6.0]	[3.0-6.0]	[4.0-5.5]	[3.0-6.0]	[3.0-5.0]	[3.0-6.0]	[3.0-6.5]
SLTs	4.0<br>[3.0-5.0]	6.0<br>[4.0-6.0]	5.0<br>[4.0-6.0]	6.0<br>[4.0-6.0]	4.0<br>[3.0-4.0]	5.0<br>[4.0-5.0]	5.0<br>[4.0-6.0]	4.0<br>[4.0-5.0]
Older adults	5.0<br>[4.0-7.0]	6.0<br>[4.5-7.0]	4.0<br>[3.0-6.0]	3.0<br>[2.0-4.0]	2.0<br>[1.5-4.5]	4.0<br>[3.0-4.5]	2.0<br>[1.5-2.0]	3.0<br>[1.5-4.0]
P-value	.08	.54	.68	**.03**	.07	.44	**.01**	.20
**Overall rating**								
Dietitians	6.0<br>[5.0-6.0]	5.0<br>[4.0-6.0]	5.0<br>[3.0-6.0]	4.0<br>[3.5-5.5]	5.0<br>[4.0-6.0]	4.0<br>[2.5-6.0]	4.0<br>[2.5-6.0]	3.0<br>[2.5-5.5]
SLTs	4.0<br>[3.8-5.3]	5.0<br>[4.0-6.0]	5.0<br>[5.0-6.0]	5.0<br>[4.0-5.0]	4.0<br>[4.0-4.0]	5.0<br>[4.0-5.0]	5.0<br>[3.0-5.0]	5.0<br>[3.0-5.0]
Older adults	5.0<br>[4.5-7.0]	7.0<br>[5.5-7.0]	4.0<br>[3.0-6.5]	3.0<br>[2.0-5.0]	2.0<br>[1.5-3.5]	4.0<br>[2.5-4.5]	2.0<br>[1.0-2.5]	3.0<br>[1.5-4.0]
P-value	.10	.07	.44	.24	**.005**	.39	**.02**	.23
**Meeting expectations**								
Dietitians<br>% (n)	89 (8)	67 (6)	56 (5)	56 (5)	56 (5)	56 (5)	44 (4)	33 (3)
SLTs<br>% (n)	29 (2)	71 (5)	86 (6)	86 (6)	57 (4)	57 (4)	57 (4)	86 (6)
Older adults<br>% (n)	80 (4)	100 (5)	60 (3)	60 (3)	20 (1)	60 (3)	0 (0)	20 (1)
P-value	.12	.31	.12	.12	.31	.99	.21	.20
**Appropriate to the described level**								
Dietitians<br>% (n)	11 (1)	44 (4)	78 (7)	22 (2)	78 (7)	44 (4)	56 (5)	78 (7)
SLTs<br>% (n)	29 (2)	43 (3)	71 (5)	43 (3)	100 (7)	14 (1)	43 (3)	76 (6)
Older adults<br>% (n)	80 (4)	100 (5)	100 (5)	100 (5)	100 (5)	100 (5)	80 (4)	80 (4)
P-value	**.04**	.10	.53	**.04**	.23	.05	.75	.93

Abbreviation: SLT = Speech Language Therapist.

Sensory aspects were rated on a 7-point scale, 1 = extremely poor, 7 = excellent. Expectation and appropriateness questions were answered by “yes, no or not sure”.

A Kruskal-Wallis test was performed to test the significance between stakeholder groups. Chi-square tests were performed to compare the categorical variables. $p<.05$ indicates significance.
